# Biosensor Design for the Detection of Circulating Tumor Cells Using the Quartz Crystal Resonator Technique

**DOI:** 10.3390/bios13040433

**Published:** 2023-03-29

**Authors:** Raad A. Alawajji, Zeid A. Nima Alsudani, Alexandrus S. Biris, Ganesh K. Kannarpady

**Affiliations:** 1 Center for Integrative Nanotechnology Sciences, University of Arkansas at Little Rock, 2801 South University Avenue, Little Rock, AR 72204, USA; raad.abdulnabi@uobasrah.edu.iq (R.A.A.); mrzeid@gmail.com (Z.A.N.A.); asbiris@ualr.edu (A.S.B.); 2 Department of Physics, College of Science, University of Basrah, Basrah 61004, Iraq

**Keywords:** quartz crystal resonator, sensing circulating tumor cells, point-of-care diagnosis, MCF-7, PANC-1, PC-3

## Abstract

A new mass-sensitive biosensing approach for detecting circulating tumor cells (CTCs) using a quartz crystal resonator (QCR) has been developed. A mathematical model was used to design a ring electrode-based QCR to eliminate the Gaussian spatial distribution of frequency response in the first harmonic mode, a characteristic of QCRs, without compromising the sensitivity of frequency response. An ink-dot method was used to validate the ring electrode fabricated based on our model. Furthermore, the ring electrode QCR was experimentally tested for its ability to capture circulating tumor cells, and the results were compared with a commercially available QCR with a keyhole electrode. An indirect method of surface immobilization technique was employed via modification of the SiO_2_ surface of the ring electrode using a silane, protein, and anti-EpCAM. The ring electrode successfully demonstrated eliminating the spatial nonuniformity of frequency response for three cancer cell lines, i.e., MCF-7, PANC-1, and PC-3, compared with the keyhole QCR, which showed nonuniform spatial response for the same cancer cell lines. These results are promising for developing QCR-based biosensors for the early detection of cancer cells, with the potential for point-of-care diagnosis for cancer screening.

## 1. Introduction

Point-of-care (POC) diagnostics is gaining significant attention in the healthcare industry due to its easy access, low cost, portability, and fast turnaround time [1]. Sensors developed based on platforms, such as micro/nano electro-mechanical systems (MEMS/NEMS), and Quartz Crystal Resonators (QCRs), are the backbone of such POC tools. The recent global pandemic SARS-CoV-2 has increased the importance of POC tools, which can enhance the detection, isolation, and prevention of spreading, and have proven effective in curbing the pandemic [2]. Bhaskar et al. have demonstrated enhancements in surface plasmon-coupled emission obtained from silver nanoparticles for the detection of femtomolar levels of iodide and zeptomolar levels of cortisol [3]. Improvements in nanoscience and microfluidic technologies have significantly increased the development of biosensors with highly desirable features, such as high sensitivity, selectivity, and reusability, for various applications, including nucleic acids, proteins, urinalysis, oral cancer, and cardiac troponin [4,5,6,7]. Further details on the efforts to develop advanced point-of-care tools are reviewed extensively by Bacon et al. in their recent review article [8].

Similarly, POC screening tools can significantly improve the quality of healthcare provided for terminal diseases such as cancer, where early detection is critical to a positive prognosis for the patient. Circulating tumor cells (CTCs) are believed to be the main pathway toward metastasis, which is the growth of secondary tumors from cells that detach from the primary tumor. CTCs are responsible for around 90% of cancer-related deaths [9], so developing techniques for early CTC detection can help create strategic treatment plans to counter metastatic growth and increase patient survival. In recent years, many advanced methods have been developed to detect and isolate CTCs early for successful treatment [10,11]. CTCs have different properties than normal cells. Various technologies have been applied to isolate them based on physical properties, such as deformability, size, and electric charge, and biological properties, such as surface protein expression. The most effective techniques combine physical and biological properties [12,13,14,15,16,17,18]. Recently, the plasmon resonance phenomena of gold and silver nanoparticles have led to the development of various detection techniques, such as Localized Surface Plasmon Resonance (LSPR) for cancer disease detection [19]. Generally, in LSPR devices, the sensing mechanisms include colorimetric biosensing and refractive index-based biosensing. Colorimetric biosensing functions are based on changes in the mode of nanoparticles, which lead to changes in color. This is based on the fact that GNPs can exist in both aggregate and non-aggregate forms in the solution [20,21]. Meanwhile, refractive index changes at the nanoparticle surface are detected by LSPR-based biosensing measurements [22]. Zhu et al. have noticed that malignant cells have a higher refractive index than normal cells, making LSPR a promising CTC detection technique [23]. The most significant advantages of LSPR are high sensitivity and specificity, real-time measurement, and the small sample size required to run it. On the other hand, the disadvantages are the complexity of the action, cost-effectiveness, and non-target binding. However, CTC detection remains a challenge because the concentration of CTCs is as low as one cell among millions of healthy cells. Therefore, we must overcome their detection and isolation limitations to develop convenient, affordable, and sensitive CTC detection techniques.

Quartz crystal resonators (QCRs) have become a widely used analytical tool due to their sensitivity to mass variations as small as nanograms in various fields, such as thin film thickness monitors and quartz crystal microbalances (QCMs) [24]. In recent decades, several researchers have started using their unique potential in the field of biological sensors. Redpenning et al. studied the rate of attachment of osteoblast cells, which are bone-forming cells, to QCMs in an aqueous solution. They observed a direct relationship between changes in the resonant frequency and surface area coverage to monitor osteoblast cell growth over several weeks [25]. Gryte et al. monitored the attachment and detachment of mammalian cells on metal surfaces in real time on the piezoelectrically active area of QCMs and observed that the anchoring and attachment of cells on the QCM surface caused a decrease in frequency [26]. Fredriksson et al. characterized living cells using the QCM technique and found that by monitoring both the frequency change and energy dissipation, valuable information can be gathered on the cell-surface adhesion process. They also observed that by using a serum-free medium, small clusters of cells (less than 100 cells) could be detected [27]. Additionally, they studied the cell attachment on QCMs in serum-containing media and proved that QCM could be an effective and powerful technique to monitor cell attachment and spreading, which can constitute a screening method in the biomaterials research area [28]. Atay et al. used QCM to detect high metastatic human breast cancer cells [29]. In their study, they deposited PHEMA nanoparticles on the surface of the QCM sensor to enhance its functionality with transferrin. The results showed that the sensor had high sensitivity and selectivity to discriminate MCF-7 cells from other cells they tested. In another study, Zhang et al. investigated the detection of breast cancer cells (MCF-7) in situ using QCM [30]. They immobilized chitosan and folic acid conjugate on the QCM surface, which was used as a receptor to capture MCF-7 cells. The device showed a detection limit of around 430 cells per milliliter.

**Figure 1:**
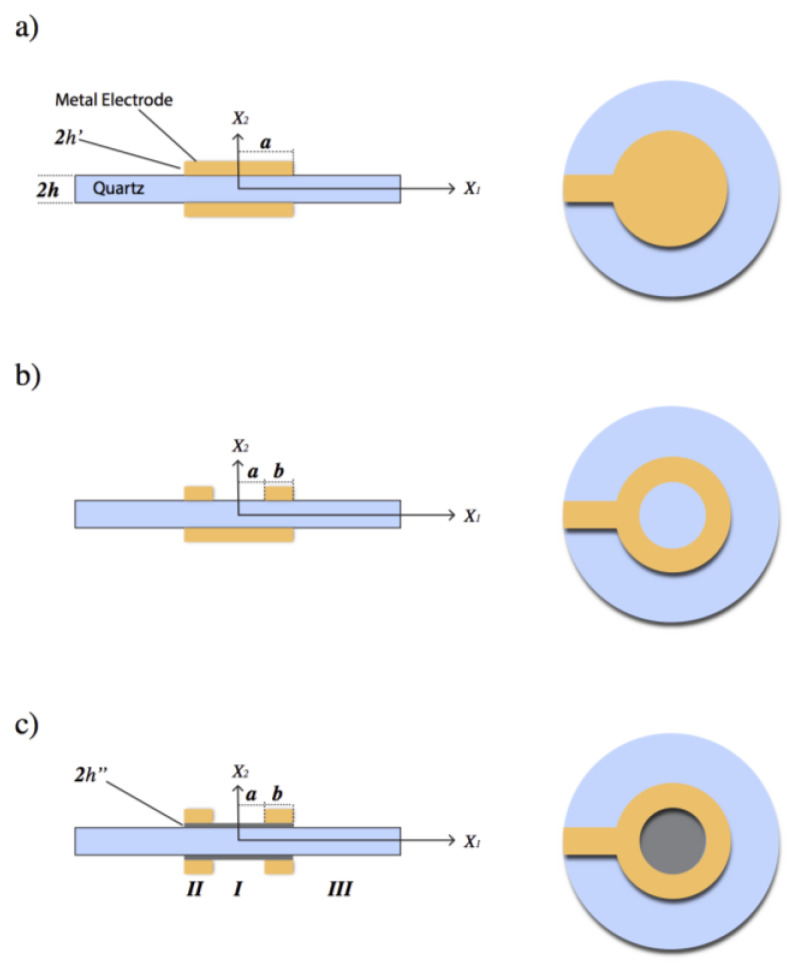
Cross section and front view of three QCR designs: (**a**) keyhole electrode QCR with radius *a*; (**b**) ring electrode QCR with inner radius a and outer radius *b*; and (**c**) modified ring electrode QCR with a first coating of solid electrodes on both sides (first layer *I*) and a second coating of ring electrodes on both sides with inner radius *a* and outer radius *b* (second metal layer *II*), and (*III*) non-electrode region.

A typical QCR is made from a disc or rectangle of quartz crystal sandwiched between two circular metallic electrodes deposited using physical vapor deposition, as shown in Figure 1. The resonators are designed to oscillate at a fundamental frequency via the piezoelectric effect when voltage is applied to the excitation electrodes and can also operate at higher frequencies (e.g., third, fifth, seventh, etc. harmonic modes) to provide higher mass sensitivity. In the 1950s, Sauerbrey theorized that adding or removing a small amount of mass from the surface of a quartz crystal electrode causes a shift in the resonance frequency. This theory established today’s well-known mass-frequency relationship used in QCRs [31].

(1) $$\Delta f\approx \frac{-2f_{o}^{2}\Delta m}{A\sqrt{\rho_{q}\mu_{q}}}{=-C}_{f}\Delta m$$

where *C_f_* is the integral sensitivity or Sauerbrey sensitivity constant (Hz m^2^/kg), Δ*f* is the frequency shift (Hz), *f_o_* is the resonant crystal frequency (Hz), Δ*m* is the mass change (kg), *A* is the active area (m^2^), *ρ_q_* is the density (kg/m^3^), and *μ_q_* is the shear modulus (N m^2^) for AT-cut quartz crystal. Equation (1) is applicable in many cases, especially with uniform thin films produced by vacuum deposition; however, it is not a sufficient rule for all conditions. Moreover, the sensitivity of QCRs shows a spatial Gaussian distribution with the highest sensitivity at the center of the resonator and the lowest at the edge of the resonator [32]. This spatial non-uniformity poses an insurmountable challenge for the potential use of QCRs in many areas, such as biological cell detection, where the attachment of the mass is random on the resonator.

To overcome the limitation of QCRs where the mass sensitivity profile reflects a Gaussian distribution, many studies have been conducted to minimize or eliminate this contribution to the sensing area. One study used an analytical model to predict the mass sensitivity profile of a decorated ring electrode on the upper side of a QCR. The model analyzed 11 MHz Plano-Plano crystals with different mass loading factors and electrode diameters. The results showed that a material with a low mass loading factor (a very thin electrode) produces a uniform mass sensitivity in the center area, but such a thin electrode layer is not practical [33,34]. The same theoretical model was used to study the mass distribution uniformity of 5 MHz crystals with a ring configuration and a large electrode area, but these dimensions caused the resonator to lose sensitivity [35]. Another approach to minimize the mass sensitivity profile involved working at a higher frequency, such as the third or fifth overtone. The results showed that higher overtones produce a nearly uniform sensing area, but also diminish mass sensitivity. The other researchers suggested that this issue could be overcome with new electrode designs, such as dot-ring or double-ring designs [36].

This study introduces two strategies to address the lack of circulating tumor cell (CTC) detection technology. We propose using a mass-sensitive device based on quartz crystal resonators (QCRs) as a promising transducer for creating a biosensor for CTC detection. First, we present a mathematical model that analyzes the performance of Plano-Plano AT-cut crystals with a fundamental frequency of 6 MHz. The model aims to minimize the Gaussian distribution by creating a ring electrode QCR to achieve a uniform mass sensitivity distribution along its radius. We conducted a comprehensive study with modifications and parameter optimization to predict the behavior of the modified resonators before fabrication. We analyzed three designs—concentric identical-electrode (keyhole), a ring electrode, and modified ring electrode QCRs—to predict their mass sensitivity distribution and determine the possibility of achieving a uniform distribution. This would allow us to estimate the spatial mass distribution when deposited mass does not cover the sensing area uniformly or completely. We then fabricated QCRs with three different electrode configurations and validated our model using an ink-dot method. In the second strategy, we further discussed the viability of the fabricated QCR designs as biosensors by modifying the surface with an anti-antibody selective layer for further investigation in CTC detection applications. We used three cancer cell lines, MCF-7, PANC-1, and PC-3, to study the frequency response of 9 MHz ring electrode QCR and compared the data with that of 6 MHz keyhole electrode QCR.

## 2. Materials and Methods

### 2.1. Mathematical Modeling of QCR Mass Sensitivity

The size, shape, and thickness of electrodes perform a crucial role in the spatial mass sensitivity of quartz crystal resonators (QCR). However, changes in electrode configuration can affect the quality factor, potentially reducing the usefulness of QCRs as sensors. The objective is to find an optimal thickness of electrodes through mathematical modeling that strikes a balance between a reasonably high-quality factor and minimal non-uniformity in radial mass sensitivity. Equation (1) is valid when the added mass is very small compared to the total mass of the crystal; in other words, when Δ*f* << *f_o_*. In addition, the homogeneous layer must cover the entire effective area in order to produce a change in mass (Δ*m*) [34]. In this case, the QCR is considered an infinite plate vibrating in the fundamental thickness shear mode (TSM) with equal amplitude and phase at every point of the quartz plate surface. However, for many applications—such as electroplating, corrosion processes, and some biological events—the added mass is not uniform and does not cover the effective electrode area completely or uniformly. As a result, the vibration amplitude distribution is not homogenous over the electrode area but instead reflects a Gaussian distribution. Therefore, the maximum vibration amplitude and, thus, the mass sensitivity will be in the center of the electrode region due to the energy trapping effect. This value decreases towards the edges of the electrode [35,36]. In other words, the frequency response would be higher for the same mass if the mass is attached to the center of the QCR electrode rather than the edge.

The Sauerbrey equation states that the integral sensitivity (*C_f_*) can be calculated by integrating the differential sensitivity function (*S_f_*) across the overlapping electrode area, as follows:

(2) $$S_{f}=\frac{{\left|A_{1}(r)\right|}^{2}}{2\pi \int_{0}^{2\pi }r{\left|A_{1}(r)\right|}^{2}}C_{f}$$

The differential sensitivity function (*S_f_*) represents the mass sensitivity expressed in Hz/kg. *A*_1_(*r*) represents the particle displacement amplitude on the surface, and *r* denotes the distance from the center of the crystal. The dependence of *S_f_* is solely on the radial distance *r* from the center, and it is unaffected by the angular distribution of the crystal plane. Therefore, one must determine the crystal’s particle displacement amplitude *A*_1_(*r*) to calculate the mass sensitivity.

Consider a two-dimensional AT-cut quartz wafer with a thickness of 2*h* in the *x*_2_ direction and extending infinitely in the *x*_3_ direction. Figure 1 shows metal electrodes of 2*h′* thickness are coated on a limited portion of the AT-cut quartz wafer. Due to the different mechanical constants (elastic and inertial) in the electrode region (*I*), the overlap of the upper and lower electrodes in the partial electrode region (*II*), the electrode tab, and the no-electrode region (*III*), the bar crystal exhibits varying characteristics. As a result, three distinct cut-off frequencies arise on the coated quartz crystal: the cut-off frequencies of the no-electrode region (*III*), the electrode region (*I*), and the region *II* beneath the electrode tabs in the partial electrode region [25]. Despite the electrodes being coated with a very thin metal, these cut-off frequencies, designated $\omega_{c}^{u}$, $\omega_{c}^{e}$, and $\omega_{c}^{p}$, are close to each other in each region [26]. Applying an electric field in the *x*_2_ direction of an AT-cut crystal will cause particle displacement in the *x*_1_ direction, coupled with the electric field. This, in turn, produces a standing wave in the *x*_3_ direction. However, TSM is not the only excited mode in quartz crystals. Other modes, such as face shear FS_1_ and thickness twist TT_3_, will also be excited, which propagate in planes *x*_1_ and *x*_3_, respectively [23]. According to the thickness-shear approximation, when the driving frequencies are very close to the TSM frequency and the wavenumber is sufficiently small, TSM dominates, and other modes make an extremely small contribution. Consequently, only the dominant mode of vibration, TSM, will be considered for this problem (assuming very small piezoelectric coupling) [24]. At a specific excitation frequency, for time-harmonic waves, the standing wave equation can be described as follows [25]:

(3) $$A_{1}\left(x_{1},x_{2},x_{3},t\right)=A_{1}\left(x_{1},x_{3}\right)sin\left(k_{2}x_{2}\right)e^{j\omega t}$$

where *k*_2_ is the shear horizontal acoustic wave number in the *x*_2_ direction, and *ω* is the angular excitation frequency (*ω* = 2*πf*). The acoustic wave equation can be simplified as follows [20,21]:

(4) $$\left(\frac{C_{11}}{C_{44}}\right)\frac{\partial^{2}A_{1}}{\partial x_{1}^{2}}+\left(\frac{C_{55}}{C_{66}}\right)\frac{\partial^{2}A_{1}}{\partial x_{3}^{2}}+\left[k^{2}-k_{2}^{2}\left(\frac{{\overset{-}{C}}_{66}}{C_{66}}\right)\right]A_{1}=0$$

where *C_ij_* is the elastic stiffness constant, k=ω/v is the wavenumber of driving frequency, and *v* is the velocity for the propagation of the shear wave given by (*C*_66_*/ρ_q_*) ^½^. ${\overset{-}{C}}_{66}=C_{66}+e_{26}^{2}/\varepsilon_{22}$ represents the acoustically stiffened elastic constant. By solving Equations (3) and (4), the resonance condition can be found for the electrode region using the following equation [33,34]:

(5) k2hk262+R2h2k22=sink2hcosk2h

where $k_{26}^{2}=e_{26}^{2}/{\overset{-}{C}}_{66}\varepsilon_{22}$ is the electrochemical coupling constant and $R=(2h^{\prime }\rho^{\prime }/h\rho )$ is the electrode mass loading factor.

The angular resonance frequency $\omega_{o}^{e}$ for the electrode region can be found by solving Equation (5) for *k_2_*, as follows:

(6a) $$\omega_{o}^{e}=\frac{\pi }{2h}{\left(\frac{{\hat{C}}_{66}^{E}}{\rho }\right)}^{½}$$

The angular resonance frequencies $\omega_{o}^{p},\omega_{o}^{u}$ on both the partial and non-electrode regions can be obtained by the same method.

(6b) $$\omega_{o}^{p}=\frac{\pi }{2h}{\left(\frac{{\hat{C}}_{66}^{P}}{\rho }\right)}^{1/2}$$

(6c) $$\omega_{o}^{u}=\frac{\pi }{2h}{\left(\frac{{\overset{-}{C}}_{66}}{\rho }\right)}^{½}$$

where ${\hat{C}}_{66}={\overset{-}{C}}_{66}(1-2R-\frac{8k_{26}^{2}}{\pi^{2}})$ is the piezoelectrically stiffened effective elastic constant. For AT-cut quartz crystal, the electromechanical coupling factor is very small $\left(k_{26}^{2}\approx 0.8\%\right)$. Therefore, the piezoelectric effect of the electrode is negligible compared to the mass loading effect when $R\gg \frac{4k_{26}^{2}}{\pi^{2}}$ [37]. According to the latter relation of ${\hat{C}}_{66}$, increasing the mass loading factor and reducing the piezoelectrically stiffened effective elastic constant make the resonance frequency, particularly the cut-off frequency, of the electrode region lower than that of both the partially coated and uncoated regions.

For convenience, Equation (4) can be converted from the Cartesian coordinate system to the polar coordinate system (*r*,*θ*) to match the boundary conditions of a cylindrical resonator. Therefore, the particle displacement amplitude across the QCR can be described in cylindrical coordinates by applying a scalar Helmholtz wave equation [37,38].

(7) $$r^{2}\frac{\partial^{2}A_{1}(r,\theta )}{\partial r^{2}}+r\frac{\partial A_{1}(r,\theta )}{\partial r}+\frac{\partial^{2}A_{1}(r,\theta )}{\partial \theta^{2}}+{\left(rk_{r}\right)}^{2}A_{1}\left(r,\theta \right)=0$$

The first two terms in Equation (7) show variation along the radial direction (*r*), which represents Bessel’s differential equation, whose solution can be given by Bessel functions. While the third term shows variation in the angular direction (*θ*), which represents angular function equation, whose solution can be given by harmonic functions. Therefore, the general solutions of Equation (7) will be in the form [33,39].

(8) $$A_{1}\left(r,\theta \right)=\left\{\begin{array}{c} \sum_{n=0}^{n=\infty }\left[C1J_{n}\left(k_{r}r\right)+C2N_{n}\left(k_{r}r\right)\right]\left[C3cos\;n\theta +C4sin\;n\theta \right]\\ \sum_{n=0}^{n=\infty }\left[C1I_{n}\left(k_{r}r\right)+C2K_{n}\left(k_{r}r\right)\right]\left[C3cos\;n\theta +C4sin\;n\theta \right] \end{array}\right.$$

where $k_{r}^{2}=k^{2}-k_{c}^{2}$, $k_{r}$ is the radial wavenumber, $k_{c}$ is the cutoff wavenumber given by $k_{c}^{2}=k_{2}^{2}\left(\frac{{\overset{-}{C}}_{66}}{C_{66}}\right),\;and$ *n* is the harmonic constant with values 0, 1, 2, 3,….

Note that the radial acoustic wavenumber $k_{r}$ becomes imaginary (<0) when $k<k_{c}$. This will result in an evanescent acoustic wave that exponentially decays when it is far away from the electrode region. In contrast, when $k>k_{c}$, $k_{r}$ becomes real (>0), resulting in an acoustic wave energy that spreads over the entire quartz plate and no evanescent acoustic wave.

*C*1, *C*2, *C*3, and *C*4 are unknown amplitude constants that can be found by applying the boundary condition. $J_{n}\left(k_{r}r\right)$ is the Bessel function of the first kind with order *n*, which has a finite limit as $\left(k_{r}r\right)$ reaches zero. $N_{n}\left(k_{r}r\right)$ is the second kind of Bessel function, with order *n*, and does not have a finite limit as $\left(k_{r}r\right)$ reaches zero. $I_{n}\left(k_{r}r\right)\;and\;K_{n}\left(k_{r}r\right)$ are, respectively, the first and second modified Bessel functions (order: *n*). $K_{n}\left(k_{r}r\right)$ has no finite limit, but $I_{n}\left(k_{r}r\right)$ has a finite limit as $\left(k_{r}r\right)$ nears zero.

When the QCR operates at a fundamental resonance mode, *n =* 0, the particle displacement amplitude varies in the radial direction (*r*) and does not vary in the angular direction (*θ*). Therefore, Equation (8) can be rewritten as follows:

(9) $$A_{1}\left(r\right)=\left\{\begin{array}{c} C1J_{o}\left(k_{r}r\right)+C2N_{o}\left(k_{r}r\right)\;{\left(k_{r}r\right)}^{2}>0\\ C1I_{o}\left(k_{r}r\right)+C2K_{o}\left(k_{r}r\right)\;{\left(k_{r}r\right)}^{2}<0 \end{array}\right\}$$

where *J_o_* and *N_o_* represent the Bessel function of the first and second kind with order zero, and *I_o_* and *K_o_* represent the modified Bessel function of the first and second kind with order zero. The constants *C*1 and *C*2 represent the particle amplitudes [33,34]. The correct solution can be chosen depending on the condition of the Bessel function.

As each region of the crystal has a different cut-off frequency, the radial component can be written in terms of operating frequency (*f*) and cut-off frequency (*f_c_*) for each region.

(10) $$k_{r}^{2}=\left\{\begin{array}{c} \left(\frac{\pi^{2}}{4h^{2}}\right)\left(\frac{1}{f_{66}^{2}}\right)\left[f^{2}-f_{ce}^{2}\right]\\ \begin{array}{c} \left(\frac{\pi^{2}}{4h^{2}}\right)\left(\frac{1}{f_{66}^{2}}\right)\left[f^{2}-f_{cp}^{2}\right]\\ \left(\frac{\pi^{2}}{4h^{2}}\right)\left(\frac{1}{f_{66}^{2}}\right)\left[f^{2}-f_{cu}^{2}\right] \end{array} \end{array}\right\}$$

where $f_{ce},f_{cp},\;and\;f_{cu}$ are the cut-off frequencies in full electrode, partial electrode, and non-electrode regions, respectively, while $f_{66}$ represents the cut-off frequency in the quartz crystal plate [34,37]. Using the cut-off frequency for each region, the radial components can be calculated, which in turn allows the particle displacement amplitudes on each region to be found.

Further details regarding the mathematical modeling approach used in this study can be found in Appendix A. The optimum thickness (2*h′*) of the electrodes for the ring and modified ring electrode configurations were determined by solving the homogeneous linear Equations (A3) and (A6) in Appendix A for ring and modified ring electrodes, respectively, using a MATLAB program. The amplitude constants in the equation were calculated to determine the particle displacement in each region. To ensure uniformity, the electrode thickness (mass loading), a crucial parameter, was optimized using MATLAB by adjusting the thickness and solving the equations until the plateau sensitivity was achieved within the electrode region of the resonator. The resulting optimized electrode thickness, loading factor, and amplitude constants for the ring and modified ring electrode designs of the 6 MHz resonator, and the ring electrode design of the 9 MHz resonator, are presented in Table 1.

The optimized electrode thickness obtained from the mathematical model was then applied to the quartz crystal design, which is discussed in the following sections.

### 2.2. Quartz Crystal Design for Mass Sensitivity Measurements

In this study, we used blank quartz crystal resonators that were Plano-Plano AT-cut crystals with a diameter of 13.97 mm and a fundamental frequency of 6 MHz. The crystals had beveled edges and optically polished surfaces. After cleaning with acetone, methanol, and deionized water for 5 min each, the crystals were dried with compressed nitrogen gas. The photolithography technique was used to create keyholes and ring electrode patterns on the blank crystals. Then, a DC magnetron sputtering technique was used to deposit a gold layer pre-coated with titanium to enhance adhesion. Three configurations of resonators were examined: a keyhole with a mass loading factor of *R* = 0.006, a ring electrode with a mass loading of *R* = 0.006, a gold layer thickness of 110 m, and a titanium layer with a thickness of 15 nm, and a modified ring electrode *R* = 0.0045 with a gold layer thickness of 50 nm, and titanium layer thickness of 50 nm. The identical-concentric electrode (keyhole) had a radius of 2.5 mm for the upper and lower solid electrodes. The ring electrode QCR design had a solid lower ring electrode radius of 2.5 mm and upper ring electrode radius of 1 mm (inner) and 2.5 mm (outer). The modified ring electrode had equal upper and lower ring electrodes with inner radii of 1 mm and outer radii of 2.5 mm.

The HC-48/U holder, consisting of two micro-springs, was used to hold the crystal resonators. The holder is mounted on an x-y stage, allowing the crystals to move in a precise radial direction. An ultra fine point Sharpie pen was used to place ink dots on the surface of the QCR devices. The pen was mounted on a z-stage to enable it to move vertically and apply the ink dots with consistent pressure, helping to minimize variations in dot mass. An optical microscope with a digital camera was used to monitor the size of the ink dots. Frequency measurements were taken using a QCA922 instrument with a frequency scan range of 1 MHz to 10 MHz and a resolution of 0.1 Hz at a sampling rate of 100 ms. The mass of the ink dots was measured using a microbalance with a resolution of 10 µg.

### 2.3. Mass Sensitivity Distribution Measurements

Most experimental methods that have been used to map the mass sensitivity distribution on a QCR surface, such as depositing a small metal spot or electroplating a wire tip on the crystal surface, have drawbacks [40,41]. In contrast, the ink dot technique is a simple, effective, and reliable method for studying the radial dependence of mass sensitivity on the resonator surface [32,42]. Additionally, the ink dot technique eliminates the stress and viscoelastic effect seen in other methods, so the recorded frequency shift is caused solely by the mass of the ink dots, which represents a rigid added mass.

**Figure 2:**
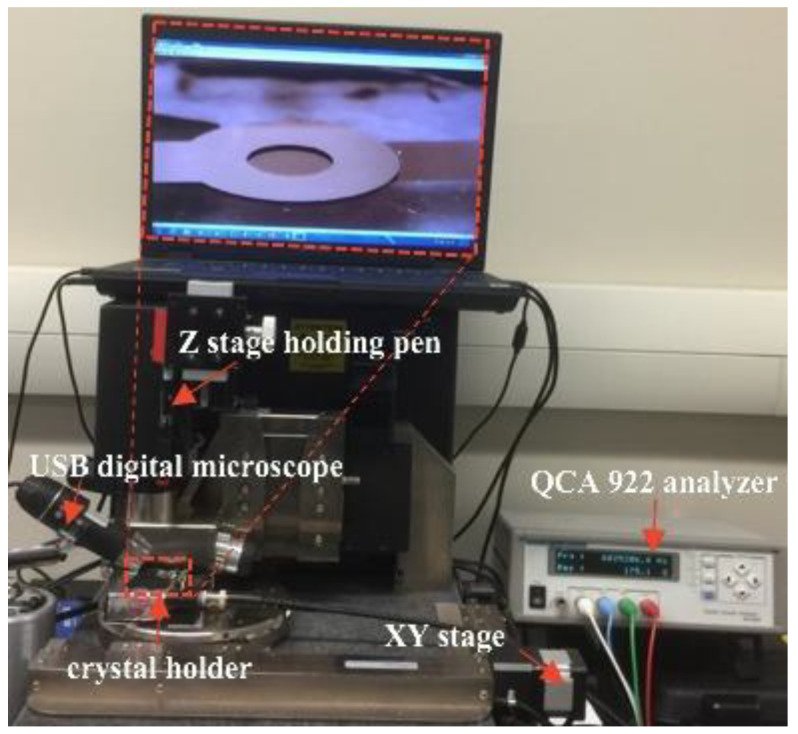
Picture of the actual set up used in the ink-dot study. An old, non-functional atomic force microscope was repurposed to utilize its motorized XYZ stage for accurately placing ink dots.

To estimate the average mass of the ink dots, 130 dots were placed on the resonators using the setup shown in Figure 2, and the total weight of the dots, as measured by the microbalance, was approximately 100 µg. Therefore, the average mass of an individual ink dot was estimated to be 0.77 ± 0.06 µg. The dot diameter was around 500 µm. To ensure accurate results, errors in dot position and mass were minimized by repeating the measurements several times at each location. The frequency shift (Δ*f*) caused by each dot at each location was then recorded in a long radial direction. The ink dots dry rapidly, so all the frequency shift measurements were carried out at the same time interval after they were deposited each time.

### 2.4. Circulating Tumor Cell Detection

#### 2.4.1. Surface Modification of the Ring Electrode

The central surface of the 9 MHz ring electrode QCR, made of SiO_2_, was modified for use as a biosensor to capture CTCs. This was completed by applying 3-Aminopropyltriethoxysilane (APTES) to the surface to aid in the immobilization of anti-antibodies. A 2% APTES solution in toluene was used to decorate the surface of the ring electrode at room temperature and incubated for 2 h in a nitrogen-rich atmosphere. Next, the surface was washed twice with the same solvent, followed by ethanol (2×), then DI water to remove any physiosorbed molecules, leaving only chemisorbed silane molecules on the surface. Finally, the surface was dried with compressed nitrogen and heated at 120 °C in an oven for 6 h under a nitrogen atmosphere to improve the quality of the APTES decoration. In order to efficiently capture cancer cells, the surface was further modified with anti-Epithelial cell adhesion molecule (EpCAM) antibodies. Two methods of applying the antibodies were studied: direct and indirect.

In the direct method, the anti-EpCAM antibody was coupled directly and covalently to the APTES-decorated ring electrode surface using a zero-length cross-linker 1-Ethyl-3-[3-dimethylaminopropyl] carbodiimide hydrochloride (EDC)/N-hydroxy succinimide (NHS) reaction. First, 10 μL of anti-EpCAM antibody (0.1 mg/mL) diluted in phosphate-buffered saline (PBS) (0.1 M, pH 7.4) was incubated with 10 μL of premixed EDC (5 μL of 0.4 mg/mL) and NHS (5 μL of 1.1 mg/mL) in 2-(N-morpholino) ethane sulfonic acid (MES) buffer solution (0.1 M, pH 4.6) to activate the carboxyl groups on the anti-EpCAM antibody. Then, EDC/NHS-activated anti-EpCAM antibody was added to the freshly prepared APTES decorated ring electrode at room temperature with gentle shaking for 2 h. Next, 1% bovine serum albumin (BSA) was added to the surface to block free sites and minimize non-specific reactions, shaking for 30 min. Finally, the surface was washed with 7PBS (2×) solution, followed by washing with DI water (2×).

In the indirect method, the anti-EpCAM antibody was coupled to the APTES decorated surface via the protein A/G, a recombinant fusion protein [16,43]. First, 10 μL of protein A/G solution (1.0 mg dissolved in 1.0 mL of 10 mM phosphate buffer, pH 7.4, and 1.0 mL of 10 mM Na-acetate buffer, pH 5.5) was activated by adding 10 μL of premixed EDC (5 μL of 4 mg/mL) and NHS (5 μL of 11 mg/mL) in MES buffer solution (0.1 M, pH 4.6) for 15 min at room temperature. Then, the EDC/NHS-activated protein A/G solution was applied to the APTES decorated ring electrode and incubated for 60 min at room temperature. This procedure allows protein A/G to be bonded covalently to the ring electrode surface. Next, excess unbonded A/G protein was removed by washing the surface with PBS solution (2×). After that, 10 μL of EDC/NHS-activated anti-EpCAM antibody (0.1 mg/mL) was applied to the surface at room temperature with gentle shaking for 1 h. Finally, the surface was washed with PBS solution (2×), followed by washing with DI water (2×).

#### 2.4.2. Surface Characterization and Cell Counting

To characterize the APTES decoration and modification of surfaces with Anti-EpCAM, we used Atomic Force Microscopy (AFM) (Model Dimension FastScan, Bruker, Billerica, MA, USA) and X-ray Photoelectron Spectroscopy (XPS) (Model K-Alpha, Thermo Scientific^TM^, Waltham, MA, USA) techniques. We obtained high-resolution XPS spectra to identify the elements present on the surfaces and estimate the composition of elements from the normalized area under the curves. AFM was used to analyze the morphology and surface RMS roughness of the ring electrode QCR sensor, both with and without surface modifications.

Three different cancer cell lines (MCF-7, PANC-1, and PC-3) were grown in separate culture flasks and maintained in DMEM supplemented with 10% FBS and 1% penicillin/streptomycin. The flasks were incubated in a humidified incubator at 37 °C with a mixture of 95% air and 5% CO_2_. Every 2 days, the cells were washed with 1× PBS and the medium was refreshed. Once the cell lines reached around 80–90% confluency, the cells were counted to be used in experiments. The media was removed, and the cells attached to the flask were rinsed twice with 1× PBS to remove dead cells. Then, 4 mL of warm trypsin (0.25%) was added to the flask and incubated for 5 min to detach the cell layer from the flask. Once the cell layer detached completely, the trypsin was neutralized by adding 8 mL of growth medium to the flask. The cells were collected in a 50-mL tube and centrifuged for 5 min at 1100 rpm until the cell pellet was visible at the bottom of the tube. The suspension (trypsin/growth medium) was aspirated from the tube, leaving the cell pellet, and the cell pellet was resuspended in 8–10 mL of fresh growth medium. Finally, to count the cells, 90 µL of the cell suspension was mixed with 10 µL of trypan blue (0.4%) using an optical microscope and hemocytometer. The cells were counted and suspended in DMEM at specific numbers before being used in the biosensor experiments. The actual number of cells attached to the QCRs was imaged using a fluorescence microscope, and ImageJ software was used to count them.

#### 2.4.3. Quartz Crystal Holder Design and Fabrication

**Figure 3:**
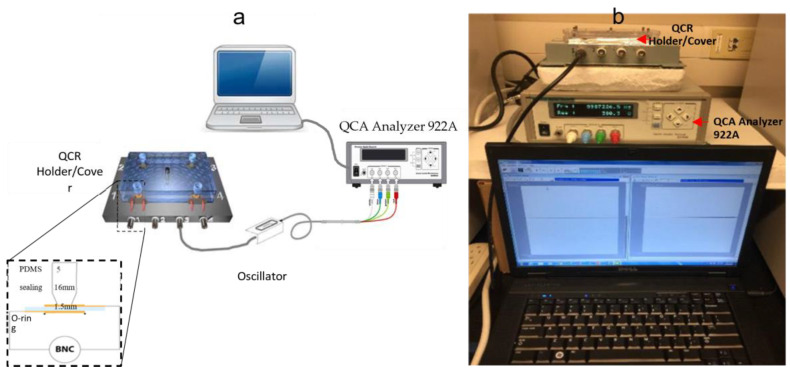
(**a**) Schematic shows the experimental setup, and the inset shows a cross-section of the keyhole QCR sensor sealed between the PDMS sealing (above) and the O-ring (below). (**b**) Picture of the actual set up used for biosensor measurements in the study.

The PDMS polymer, consisting of two components, produced a unique sealing design for confining circulating tumor cells (CTCs) in the central area of the QCR surface, 1.5 mm in diameter. The crosslinker/curing agent mix was chosen for its biocompatibility and easy formability, with a component ratio of 1:10 by weight proven to provide excellent mechanical and elastic properties. After mixing the PDMS components, the blend was degassed in a desiccator for 60 min to remove any trapped bubbles. Meanwhile, a casting mold was constructed using a self-locking microcentrifuge tube with a 2 mL volume. A hole of 1.5 mm diameter was created in the bottom of the tube by drilling, and a 200 µL tip was inserted through the hole. The PDMS mix was poured into the tube and left in the desiccator to dry for 24 h. Next, the dried PDMS was removed from the mold, cut to the required size, and annealed in an oven at 150 °C for 24 h. The steps for preparing the PDMS sealing design are shown in Figure 3. The final product was sonicated in toluene for 30 min to remove unbound molecules, then annealed for 48 h. Finally, the PDMS sealing design was mounted on the four corners of a 96-well microplate cover using epoxy adhesive, creating the final QCR cover design as shown in Figure 3.

Conducting experiments with cancer cells requires repeating the process multiple times in order to obtain reliable results and reduce errors. Therefore, a practical and cost-effective quartz crystal resonator (QCR) sensor holder is essential. To achieve this, a holder with four crystals was made, allowing multiple experiments to be conducted simultaneously under the same conditions. First, HC-48/U holders, consisting of two micro-springs, were mounted on each corner of the plastic base using quick-drying epoxy adhesive. The holder contact clips were then soldered to the male BNCs mounted on the side of the holder base. Finally, the QCR was sealed between a Teflon-coated silicone O-ring (below) and a PDMS sealing design cover (above). Figure 2 shows a representation of the final QCR base holder design.

#### 2.4.4. Biosensor Measurements

We used three cancer cell lines—MCF-7, PANC-1, and PC-3—to test the efficiency of the fabricated biosensor. These cell lines were chosen because their adhesion to the gold surface and the APTES-decorated anti-EpCAM modified SiO2 surface is strong. In each experiment, four crystals were mounted on the holder, then connected to the QCA922 analyzer to record the first reading of the frequency (*F*1) and resistance (*R*1) in the air for each crystal. Two resonators served as the control (reference), incubated with 50 μL of pure media. The other two resonators, tested for efficiency, were incubated with 50 μL containing 100–500 cancer cells. The holder was then placed inside an incubator for 30 min to allow the cells to attach to the QCR surface. After the cells were attached, they were fixed with 50 μL of 0.04% paraformaldehyde for 15 min. Next, the QCRs were washed gently with BPS (1×) once and with warm DI water (3×). The crystals were left for a couple of hours to dry, then a second reading of the frequency (*F*2) and resistance (*R*2) was recorded in the air, and the frequency/resistance shifts were calculated (Δ*F* = *F*2 − *F*1; Δ*R* = *R*2 − *R*1). In each experiment, the reference reading value was subtracted.

The attached cells were stained with Alexa Fluor 488 phalloidin and 4′,6-diamidino-2-phenylindole (DAPI) to count the number of cells attached and calculate the total area. First, the fixed cells were permeabilized in 1% Triton X-100 for 5 min, then washed three times with PBS for 5 min each. Next, to block nonspecific binding, cells fixed on the sensor were incubated with 1% BSA for 30 min, then rinsed once with PBS. Next, 200 μL (165 nM) of phalloidin was added to the cells for 60 min at room temperature in the dark, then the cells were washed twice with PBS. After that, 200 μL (300 nM) of DAPI was added to the sensor for 10 min, then rinsed with PBS and DI water twice. Lastly, the QCRs were removed from the holder for observation under a fluorescence microscope. ImageJ software was used to determine the total number of cells attached and the total coverage area on the QCR.

#### 2.4.5. Origin of Cancer Cell Lines

The human breast cancer cell line MCF-7, human pancreatic cancer cell line PANC-1, and human prostate cancer cell line PC-3 were purchased from the American Type Culture Collection (ATCC) and maintained using established procedures in liquid nitrogen.

## 3. Results and Discussion

### 3.1. Mass Sensitivity Distribution Measurements

**Figure 4:**
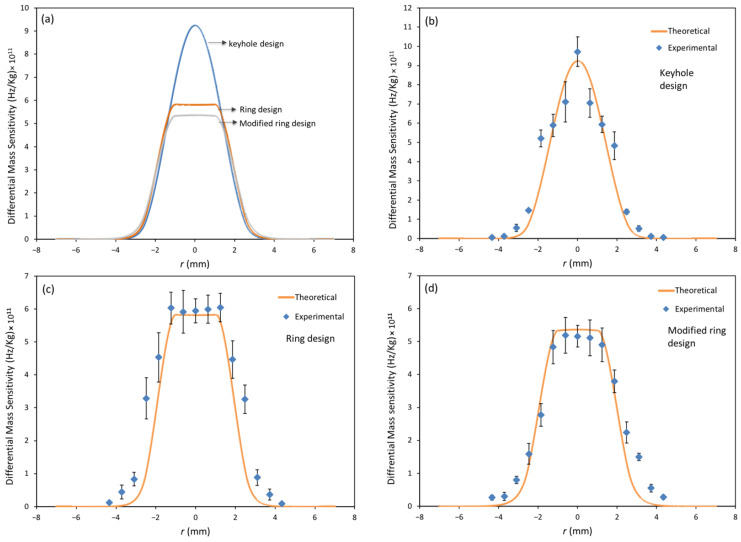
(**a**) Comparison of mass sensitivity distribution of 6 MHz Plano-Plano resonator for keyhole design (*R* = 0.006), ring electrode (*R* = 0.006), and modified ring electrode (*R* = 0.0045). Calculated and experimental data of mass sensitivity distribution as a radial location from the center of 6 MHz Plano-Plano resonator designs: (**b**) keyhole (*R* = 0.006), (**c**) ring electrode (*R* = 0.006), and (**d**) modified ring electrode (*R* = 0.0045).

Figure 4a compares the frequency response as a function of radial distance from the center of the crystal for the keyhole electrode, a ring electrode, and modified ring electrode QCRs, all using a 6 MHz Plano-Plano crystal, as obtained by our mathematical model. As expected, the keyhole QCR configuration with a mass loading factor of *R* = 0.006 had the highest mass sensitivity in the center (around 9.25 × 10^11^ Hz/kg) due to the high energy trapping in the center of the crystal. The ring electrode QCR configuration with a mass loading factor of *R* = 0.006 showed lower mass sensitivity (around 5.8 × 10^11^ Hz/kg) with a uniform mass distribution along the partial electrode region. The modified ring electrode with a mass loading factor of *R* = 0.0045 displayed slightly lower mass sensitivity (around 5.5 × 10^11^ Hz/kg) and a uniform mass sensitivity distribution in the electrode region compared to the ring electrode configuration. The maximum reduction in mass sensitivity was 37.3% and 40.5% for the ring electrode and modified ring electrode QCRs, respectively, compared to the keyhole electrode QCR.

Figure 4b–d compares the measured and predicted differential mass sensitivity of the three QCR designs as a function of radial distance. As expected for the keyhole design with a loading factor of *R* = 0.006, 110 nm gold, and 15 nm titanium, the Gaussian profile is clear, and the mass sensitivity drops gradually towards the edges of the electrode, as shown in Figure 4b. This is because the energy is mainly confined in the center of the electrode due to energy trapping. On the other hand, for the ring electrode design with the same loading factor, an inner ring diameter of 2 mm, and an outer diameter of 5 mm, the mass sensitivity in the central 2 mm produces a flat response. Similarly, the modified ring electrode shows a flat region when the mass loading is around *R* = 0.0045, as shown in Figure 4c. In other words, the center region of these designs (inner diameter: 2 mm) has the same mass sensitivity everywhere in this area because the acoustic waves have equal amplitude at each point (mass sensitivity is proportional to the square of local wave amplitude). The figure clearly shows that the measured mass sensitivity data followed the predicted trends based on our model for all designs. The slight deviation between measured and predicted data may be due to the error in measuring the exact mass and radial location of the ink dots on the resonator surface each time. Though this effect was minimized in our setup, some errors cannot be avoided. Overall, the experimental data agreed with the predicted mass sensitivity and showed the same trend. The ink dot method is affordable and accurate, making it very attractive for examining and calibrating the mass sensitivity of any QCR design.

### 3.2. Analysis of Surface Modification

The AFM and XPS analyses were conducted to confirm the proposed structure and emphasize the binding of protein A/G on the biosensor’s surface. Protein A/G was deposited on the APTES-decorated ring electrode QCR surface to facilitate attachment of the anti-EpCAM antibody to the QCR surface through Fc domains, allowing for better orientation and enhanced performance of the biosensor. The elemental percentages of the APTES-decorated, the anti-EpCAM antibody-immobilized APTES-decorated, and the protein A/G-deposited APTES-decorated ring electrode QCRs are shown in Table 2. It is clear that there is an increase in N1s and C1s elements composition, and a decrease in Si2p and O1s elements compared to the APTES-decorated ring electrode, which confirms the successful coverage of the anti-EpCAM antibody and protein A/G layers on the APTES-decorated ring electrode QCR. This is because oxygen is present in the anti-EpCAM antibody, protein A/G protein, and on the QCR surface, and carbon is a contaminant that is present on the surface of the QCR sensor. The increase in the percentage of nitrogen is considered the most important aspect in describing and confirming the formation of the anti-EpCAM antibody and protein A/G protein layers on the APTES-decorated ring electrode QCR surface.

**Figure 5:**
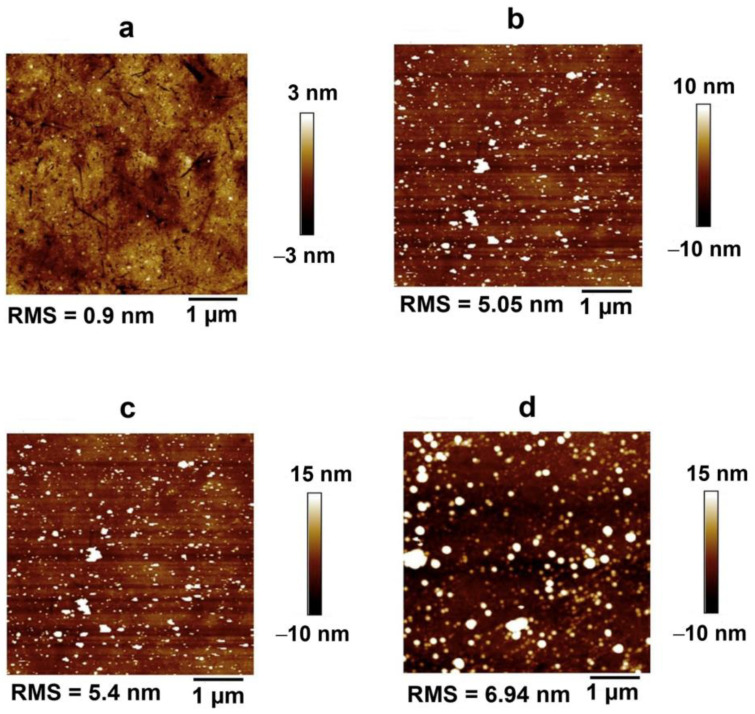
Surface morphology of QCRs, (**a**) APTES-decorated, (**b**) protein A/G on APTES-decorated, (**c**) anti-EpCAM on APTES-decorated (direct method), (**d**) anti-EpCAM on APTES-decorated with protein A/G intermediate layer (indirect method).

AFM analysis was performed using tapping mode on dry, freshly prepared samples at room temperature. The figure shows a comparison between the APTES-decorated ring electrode QCR with and without protein A/G. The surface without protein A/G had a very uniform and smooth APTES coating with a roughness of approximately 0.9 ± 0.09 nm (Figure 5a). Once protein A/G was deposited, the surface’s roughness increased rapidly to around 5.05 nm ± 0.5 (Figure 5b). This significant difference in roughness values indicates the successful coating of the surface by protein A/G. The AFM analysis also revealed the formation of multiple layers of protein A/G, and some protein aggregation higher than 10 nm based on the high section profile. However, smaller protein A/G aggregates (<10 nm) were more dominant and could be attributed to the formation of two or more protein A/G layers on the APTES-decorated surface. Previous reports [44] have indicated that the first layer of protein A/G that firmly bonds to a supported surface becomes a denatured protein layer in a short time, while the second monolayer of the protein remains non-denatured and ready to bond to the IgG.

Figure 5c,d depict the topography of anti-EpCAM antibody immobilized on an APT-ES-decorated ring electrode QCR surface using both direct and indirect methods. Both methods exhibit granular structures of anti-EpCAM of varying sizes on the surface. The indirect method, which involves using protein A/G as an intermediate layer, helps to organize EpCAM antibodies on the protein A/G deposited on the APTES-decorated ring electrode QCR, resulting in improved surface coverage compared to the direct method, which displays lower coverage quality. The roughness of the indirect method is slightly higher at RMS 6.94nm, compared to the direct method, which is approximately RMS 5.40nm. This increase in roughness, combined with the improved coverage, suggests the vertical orientation of anti-EpCAM, as previously demonstrated in a study of a similar antibody by Farris and McDonald [45].

### 3.3. Cancer Cell Detection

We chose to study the efficiency of cancer cell detection using a 9 MHz ring electrode because this electrode configuration showed the highest frequency response in ink-dot testing and flat frequency response as a function of radial distance, as described in Section 3.1. We compared the efficiency of the ring electrode QCR (9 MHz) to that of a commercially available 6 MHz keyhole electrode QCR, which is commonly used for measuring thin film thickness in physical vapor deposition. While 9 MHz QCRs have a higher intrinsic frequency than 6 MHz QCRs for a given mass change, our goal is to demonstrate the difference in the spatial distribution of frequency response between commercially available keyhole electrodes and our model-based ring electrodes. Thus, the difference in intrinsic frequencies between 6 MHz and 9 MHz QCRs does not affect the main focus of our study.

#### 3.3.1. Capture Efficiency of Anti-EpCAM Immobilized APTES Decorated Ring Electrode QCR

The performance of cell-detecting biosensors largely depends on the effectiveness of the selective layer immobilized on the biosensor surface. To enhance the ability of a QCR biosensor to capture circulating tumor cells (CTCs), it is ideal for immobilizing an anti-EpCAM antibody layer in a way that makes its active sites easily accessible for interaction with the antigens on targeted CTCs. To evaluate capture efficiency, we used MCF-7 cells as a test sample [12], which have a high expression of EpCAM, promoting binding to the immobilized anti-EpCAM antibody, for testing on APTES-decorated ring electrode QCR immobilized by direct and indirect methods.

**Figure 6:**
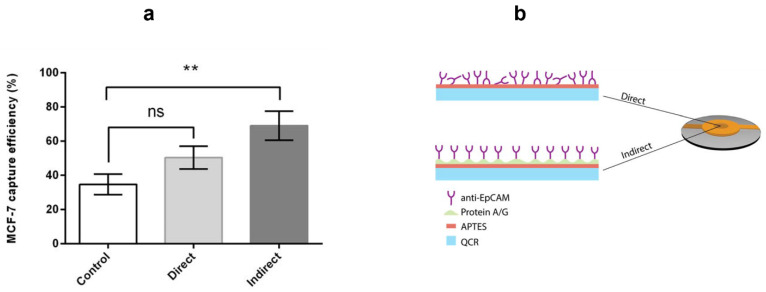
(**a**) Comparison of capture efficiencies of MCF-7 cells by control, direct, and indirect antibody immobilization strategies of APTES-modified ring electrode QCR (ns = not significant *p* > 0.05, ** = very significant 0.001 > *p* > 0.01). (**b**) Representation of direct and indirect anti-EpCAM antibody immobilization techniques of APTES-decorated 9 MHz ring electrode QCR.

Figure 6a illustrates the percentage of MCF-7 cells captured by the ring electrode QCR biosensor based on the mean values of three experiments. The control QCR, an APTES-decorated ring electrode QCR without an anti-EpCAM antibody coating, had the lowest number of attached cancer cells among the three tested QCRs. The QCR with anti-EpCAM applied using the direct method showed a slight increase in the number of captured cells, followed by the highest number of cells captured on the QCR with anti-EpCAM applied using the indirect method. The low number of captured cells on the control QCR can be attributed to non-specific interaction between the MCF-7 cells and the QCR surface. On the other hand, in the case of the direct immobilization method, many of the anti-EpCAM antibodies bond covalently to the APTES layer through antigen-binding active regions, as represented schematically in Figure 6b. As a result, the antibodies lose their activity to capture cancer cells, which can explain the lower capture efficiency. To maximize biosensor efficiency, the antibodies must maintain their activity during immobilization on solid surfaces.

The indirect immobilization method of the APTES-decorated ring electrode QCR device using protein A/G mediated anti-EpCAM antibody overcomes the limitation of random antibody orientation, and shows the highest capture efficiency. The recombinant protein A/G is a genetically engineered protein that combines the IgG binding domains of protein A and protein G, which are extracted from the surfaces of staphylococci and streptococci [46,47]. The protein A/G specifically binds to the Fc domain of various subclasses of IgG, and the advantage of using this protein mixture compared to individual proteins A or G is that it covers almost the whole range of the subclasses of IgG and is less sensitive to pH variations. Therefore, this protein mixture is bonded covalently to the APTES-decorated QCR biosensor to help maintain the activity of the anti-EpCAM antibody. Increasing the efficiency of the QCR biosensor coated by the indirect method confirms that the anti-EpCAM antibody layer is well-oriented on the sensing area of the QCR biosensor, as shown in Figure 6b, which promotes antibody-antigen binding. The well-orientation of the anti-EpCAM antibodies is related to the strong affinity bonding between the Fc domains of the anti-EpCAM antibodies and the protein A/G deposited on the biosensor surface. This facilitates the attachment of MCF-7 cells to the biosensor surface through antibody-antigen interaction, resulting in the highest capture efficiency. It is crucial to control the orientation of the anti-EpCAM antibody on the biosensor’s surface to maximize cell capture efficiency. In the case of the indirect method, the paratope (antigen-binding site) on the surface of the immobilized antibody is openly accessible to the epitope (binding site) of the MCF-7 cells that overexpress the EpCAM transmembrane protein. This promotes the binding of the cells, thereby maximizing capture effectiveness. On the other hand, in the direct method, the orientation of the anti-EpCAM antibody is random, which means that the antigen-binding site on the antibody’s surface is not freely accessible. As a result, this obstructs the binding of the MCF-7 cells to the biosensor, leading to lower capture efficiency [48].

#### 3.3.2. Spatial Sensitivity Measurements

**Figure 7:**
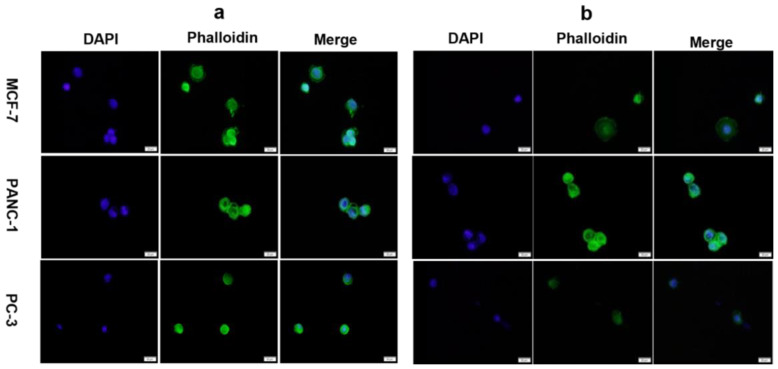
Fluorescence images of three different cancer cell lines attached and fixed to (**a**) 6 MHz keyhole QCR and (**b**) 9 MHz ring electrode QCR with an incubation time of 30 min and a magnification of 40×. The images represent a random area (scale of 20 microns) on the active regions of the quartz crystals and do not represent the total number of captured cells.

Based on our findings, we studied the frequency response of the APTES-decorated ring electrode QCR using an indirect method for immobilizing anti-EpCAM for three cancer cell lines as they had the potential to capture the highest number of cancer cells. We also examined the frequency response of the keyhole QCR for the same three cancer cell lines and compared the results with the ring electrode to evaluate the effectiveness of our electrode design. Figure 7 displays representative fluorescence images of attached cancer cells. We captured several fluorescence images for each cell line for each design and used them to count the number of cells attached to the surfaces.

**Figure 8:**
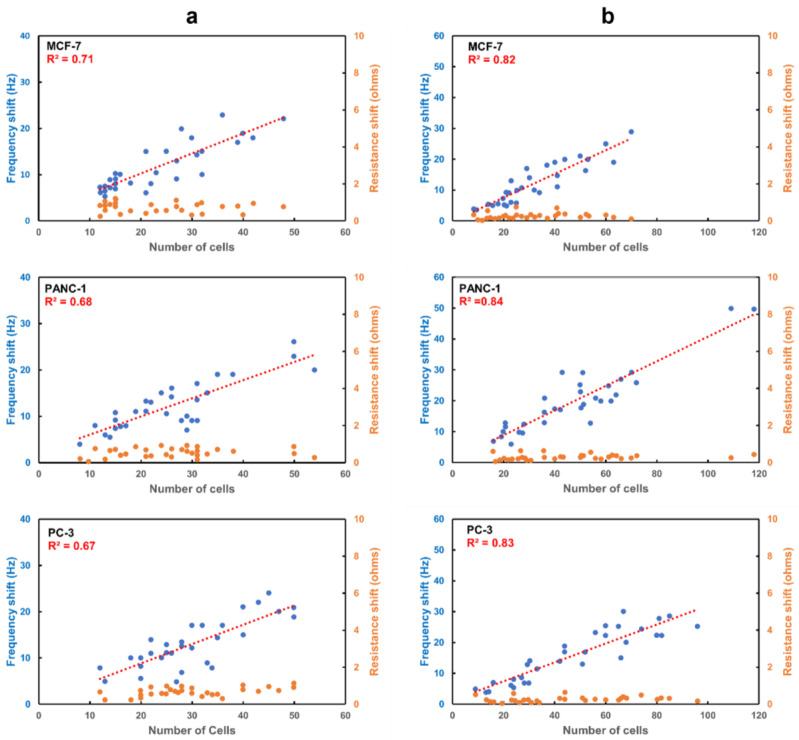
Frequency shift as a function of the number of attached cells on (**a**) 6 MHz keyhole electrode QCR and (**b**) 9 MHz ring electrode QCR for three different cancer cell lines, *n* = 30.

Figure 8 illustrates the frequency shift as a function of the number of captured cells for the three cell lines for both electrode designs. The frequency shift of both designs is proportional to the number of attached cells, as an increase in the number of attached cells increases the frequency shift. The average frequency shift caused by a single cell in the case of the 6 MHz keyhole QCR was approximately 0.39 ± 0.09 Hz, 0.42 ± 0.10 Hz, and 0.32 ± 0.08 Hz for the MCF-7, PANC-1, and PC-3 cell lines, respectively. Similarly, the average frequency shift caused by a single cell was around 0.48 ± 0.12 Hz, 0.49 ± 0.17 Hz, and 0.46 ± 0.11 Hz for the MCF-7, PANC-1, and PC-3 cell lines, respectively, in the case of the APTES-decorated 9 MHz ring electrode QCR. The value of R^2^, which measures the goodness of the linear regression fit, was found to be much higher for the APTES-decorated 9 MHz ring electrode QCR, ranging from 0.82 to 0.84 for different cell lines, compared to 0.67 to 0.71 for the 6 MHz keyhole QCRs, respectively.

The significantly higher R^2^ value indicates that the spatial variation in the mass of the cells is minimized for the APTES-decorated 9 MHz ring electrode QCR. As a result, the frequency change is caused only by the added mass, and there is no effect due to the location of the CTCs on the surface of the ring electrode QCR. In the case of the keyhole design, the significantly lower R^2^ was likely caused by two factors: the different cell size distribution and the random location of the cells on the surface of the keyhole QCR. However, it is safe to say that the lower R^2^ is mainly due to the spatial nonuniformity of the keyhole electrode design. As seen in Figure 7, the size of the captured cancer cells is comparable in both electrode designs, i.e., the keyhole and the ring electrode designs. From our ink dot experiments in Section 3.1, it was observed that the keyhole electrodes showed a Gaussian spatial distribution of frequency response for the exact size of ink dots along the axis of the QCR. It is well-known that the sensitivity of the keyhole QCR is very high in the central area of the electrode due to energy trapping, and any shift from the center will cause a significant variation in frequency. Therefore, when CTCs are attached very close to the center of the electrode, a higher frequency response is expected compared to CTCs attached at a distance away from the center of the electrode, which will produce a lower frequency response. For both designs, the resonance resistance shift (Δ*R*) is negligible and does not show an increase with an increase in the number of attached cells. This trend in resonance resistance confirms that fixed CTCs to the QCR surface behave as rigid and non-viscous, indicating the frequency shift is caused only by the mass of the CTCs.

Therefore, it can be concluded that our model-based ring electrode successfully eliminates the spatial nonuniform frequency response and has enormous potential in commercializing POC tools for early cancer detection. This result is significant because we achieved spatial uniformity of frequency distribution without compromising the frequency sensitivity by using the first harmonic modes of the QCR in comparison to some other works that used higher harmonic modes, such as third, fifth, and seventh, which reduces the frequency sensitivity. Table 3 provides a summary of the various techniques that have been used to identify different types of circulating tumor cells (CTCs). The table includes the upper limits of detection previously published in the literature and the recognition receptors used by biosensors targeting CTCs. Our ring design QCR was successful in detecting as few as 10 cells, which is lower than other techniques in general, and, in particular, quartz crystal-based sensors. Although more studies are required to fine-tune the design due to the complexity involved in detecting cancer cells at an early stage, such as the rapid growth of cancer cells and splitting of cancer cells, the ring electrode design is the first step in such an endeavor.

## 4. Conclusions

In summary, we have demonstrated the detection of circulating tumor cells (CTC) using quartz crystal resonators (QCRs), which could change the way cancer screening is performed in the future. The mathematical model was employed to predict the mass sensitivity behavior of commercially available keyhole electrode QCRs, and it was compared to our designs of the ring electrode and modified ring electrode QCRs. The model was used to optimize the thickness of the electrodes, which is crucial in confining the acoustic wave propagation within the electrode area, thus eliminating spatial non-uniformity in the first harmonic mode of QCRs. This is a significant improvement over previous works that achieved uniform spatial mass sensitivity at higher harmonic modes, but did so at the cost of a lower quality factor, which reduced overall sensitivity. We tested the ring electrode QCR for its ability to detect three different cancer cell lines by measuring the uniform spatial frequency shift. An indirect method of modifying the surface of the QCR using protein A/G-mediated anti-EpCAM antibodies was developed to maximize the capture of cancer cells, which is a crucial factor in increasing the frequency shift. The results showed that our ring electrode QCRs performed better than commercially available keyhole electrodes for all three cancer cell lines tested with a detection sensitivity of as few as 10 cells.

## Figures and Tables

**Table 1:** A list of optimal results for different electrode configurations as determined by mathematical modeling.

Electrode Design	Inner Radius *a* (mm)	Outer Radius *b* (mm)	Gold Electrode Thickness (nm)	Titanium Electrode Thickness (nm)	Mass Loading	Amplitude Constants			
						*C*1	*C*2	*C*3	*C*4
6 MHz-ring	1	2.5	110	15	0.0060	−0.069	−0.085	−0.038	−0.993
6 MHz-modified ring	1	2.5	50	50	0.0046	−0.124	−0.152	−0.060	−0.978
9 MHz-ring	1	2.5	35	15	0.0031	−0.027	−0.033	−0.017	−0.998

**Table 2:** Elemental composition calculated by XPS analysis.

	Elemental Composition (Atomic %)			
QCRs	Si	O	C	N
APTES	30.05 ± 0.3	55.48 ± 0.63	12.19 ± 0.83	2.28 ± 0.14
Anti-EpCAM	20.89 ± 0.82	39.91 ± 1.23	31.67 ± 1.68	7.52 ± 0.43
Protein A/G	14.65 ± 0.79	30.96 ± 1.36	44.86 ± 2.05	9.54 ± 0.29

**Table 3:** Table comparing the detection efficiency of various techniques, including quartz crystal-based sensors, with our work.

Technique	Recognition Element	Sensitivity of Detection	Targeted Cells	Reference
Quartz crystal microbalance biosensor	transferrin	500 cells	MDA-MB 231, MCF-7	[29]
Quartz crystal microbalance biosensor	Folic acid (FA)	430 cells	MCF-7	[30]
Quartz crystal microbalance biosensor	ALT04 antibody	100 cells	LCC	[49]
Au nanoparticle-based colorimetric method	HER2	100 cells	SK-BR-3	[50]
Quartz crystal microbalance biosensor	Notch-4 receptor antibody	42 cells *	MDA MB 231	[51]
Electrochemical impedance spectroscopy	Anti-EpCAM antibody	10 cells	MCF-7	[52]
Ring electrode QCR biosensor	Anti-EpCAM antibody	10 cells	MCF-7, PANC-1, and PC-3	this study

* Per experimental data presented in the paper.

## Data Availability

The data presented in this study are available upon request from the corresponding author.

## Appendix A

### Appendix A.1. Keyhole Electrode QCR

The structure of the concentric identical-electrode QCR (m-m) is considered the simplest and most common configuration of QCR devices (Figure 2). The m and m represent the upper and lower solid electrodes, which have the same diameter. In this QCR configuration, only electrode and non-electrode regions exist. The excited acoustic wave on the crystal surface is largely confined within the electrode region (0 ≤ *r* ≤ *a*), so the convenient operating frequency will fall between cut-off frequencies on non-electrode and fully electrode regions, i.e., $f_{cu}>f>f_{ce}$.

By simplifying Equation (9), the particle displacement in each region can be found using the following equation:

(A1) $$A_{1}\left(r\right)=\left\{\begin{array}{c} C1J_{o}(k_{r}^{e}r)\;0\leq r\leq a\\ C2K_{o}\left(k_{r}^{u}r\right)\;a\leq r\leq \infty \end{array}\right\}$$

During vibration, the central particle displacement amplitude in the center of the electrode region is assigned a finite value (*A*_1_) that represents the maximum vibration. At the center (*r =* 0), the Bessel function of the second kind (*N_o_*) is undefined by virtue of its singularity, and, therefore, it is discarded as a solution to Equation (9). Hence, *C*2 = 0. As the resonator is clamped at the edge, the particle displacement amplitude will vanish at the edge of the resonator. Thus, in the non-electrode region, the modified Bessel function of the first kind (*I_o_*) cannot be a solution to Equation (9) because the function is undefined by virtue of its singularity (goes to infinity) when *r →* ∞. Hence, *C*1 = 0 [39,53].

The remaining *C*1 and *C*2 in Equation (A1) are the particle displacement amplitude constants and can be found by applying the following boundary conditions in each region: (1) the continuity of the particle displacement *A*_1_ and (2) the continuity of the shear strain field $\frac{\partial A_{1}}{\partial r}$ at *r = a* for the electrode and non-electrode regions.

### Appendix A.2. Ring Electrode QCR

The ring electrode QCR structure consists of a lower solid electrode with radius *b* and an upper ring electrode with inner and outer radii *a* and *b*, respectively. This QCR configuration includes three regions: fully electrode, partially electrode, and non-electrode, as shown in Figure 2. Depending on the operating frequency conditions, the excited acoustic waves either exist within both the electrode and partially electrode regions $f_{cu}>f>f_{cp}>f_{ce}$, or are confined to the electrode region $f_{cu}>f_{cp}>f>f_{ce}$.

By simplifying the solution of Equation (9), we get the following [33]:

(A2) $$A_{1}\left(r\right)=\left\{\begin{array}{ll} C1I_{o}\left(k_{r}^{p}r\right) & 0\leq r\leq a\\ C2J_{o}\left(k_{r}^{e}r\right)+C3N_{o}\left(k_{r}^{e}r\right) & a\leq r\leq b\\ C4K_{o}\left(k_{r}^{u}r\right) & b\leq r<\infty \end{array}\right\}$$

In the partially electrode region, the modified Bessel function of the second kind (*K_o_*) is excluded because the function has singularity at the origin (*r* = 0). The modified Bessel function of the first kind (*I_o_*) has been discarded in the non-electrode region because the Bessel function *I_o_ →* ∞ when *r →* ∞. In the electrode region, Bessel functions of both the first and second kinds exist (a≤r≤b). By applying the same boundary conditions as for the identical-electrode QCR (m-m), Equation (A2) will yield four linear homogenous equations, which can be rearranged as a matrix:

(A3) $$\left[\begin{array}{cccc} I_{o}\left(k_{r}^{p}a\right) & -J_{o}\left(k_{r}^{e}a\right) & -N_{o}\left(k_{r}^{e}a\right) & 0\\ k_{r}^{p}I_{1}\left(k_{r}^{p}a\right) & k_{r}^{e}J_{1}\left(k_{r}^{e}a\right) & k_{r}^{e}N_{1}\left(k_{r}^{e}a\right) & 0\\ 0 & J_{o}(k_{r}^{e}b) & N_{o}\left(k_{r}^{e}b\right) & -K_{o}\left(k_{r}^{u}b\right)\\ 0 & -k_{r}^{e}J_{1}\left(k_{r}^{e}b\right) & -k_{r}^{e}N_{1}\left(k_{r}^{e}b\right) & k_{r}^{u}K_{1}(k_{r}^{u}a) \end{array}\right]\left[\begin{array}{c} C1\\ C2\\ C3\\ C4 \end{array}\right]=0$$

Equation (A3) yields a nontrivial solution when the determinant of the matrix vanishes. The matrix of the particle amplitude constants (*C*1, *C*2, *C*3, and *C*4) cannot be a zero trivial solution; therefore, the matrix on the left must equal zero. Using these parameters, the particle amplitude constants (*C*1, *C*2, *C*3, and *C*4) can be found and utilized to calculate the radial dependence of differential mass sensitivity (Equation (2)).

### Appendix A.3. Modified Ring Electrode QCR

In this design, the upper and lower electrodes have the same dimensions as ring electrodes. The structure of the modified ring electrode QCR consists of three regions. The first region (*I*) is fully electrode in the center of the QCR and has one metal layer (*Ti*), the second region (*II*) is fully electrode with two different metal layers (*Ti/Au*), and the third region (*III*) is non-electrode, as shown in Figure 2. The cut-off frequencies of these regions can be defined as *f_ceI_*, *f_ceII_****_,_*** and *f_cu_**_,_*** respectively. The excited acoustic waves could exist in any region depending on the operating frequency conditions; herein, the acoustic wave will be confined to electrode regions *I* and *II*, so the following condition applies: $f_{cu}>f>f_{ceI}>f_{ceII}$.

The mass loading factors for both regions can be given by the following equation:

(A4a) $$R_{I}=\left(\frac{2h^{\prime \prime }\rho^{\prime \prime }}{h\rho }\right)$$

(A4b) $$R_{II}=\left(\frac{2h^{\prime }\rho^{\prime }}{h\rho }\right)+\left(\frac{2h^{\prime \prime }\rho^{\prime \prime }}{h\rho }\right)$$

(A4c) $$R=R_{I}+R_{II}$$

where ${R_{I}\;and\;R}_{II}$ are the mass loading factors of regions *I* and *II*, respectively, ${{2h}^{\prime \prime },\rho }^{\prime \prime },{2h}^{\prime }{,\;and\;\rho }^{\prime }$ are the thickness and density of regions *I* and *II*, respectively. Herein, R represents the total mass loading factor in both regions (*I* and *II*) for a modified ring electrode.

The solution for this configuration is as follows:

(A5) $$A_{1}\left(r\right)=\left\{\begin{array}{ll} C1J_{o}\left(k_{r}^{eI}r\right) & 0\leq r\leq a\\ C2J_{o}\left(k_{r}^{eII}r\right)+C3N_{o}\left(k_{r}^{eII}r\right) & a\leq r\leq b\\ C4K_{o}\left(k_{r}^{u}r\right) & b\leq r<\infty \end{array}\right\}$$

where $k_{r}^{eI},k_{r}^{eII},\;and\;k_{r}^{u}$ are the radial components for regions *I*, *II*, and *III*, respectively.

According to Equation (A5), and by applying the boundary conditions, four linear homogenous equations are produced and can be rearranged as a matrix:

(A6) $$\left[\begin{array}{cccc} J_{o}\left(k_{r}^{eI}a\right) & -J_{o}\left(k_{r}^{eII}a\right) & -N_{o}\left(k_{r}^{eII}a\right) & 0\\ -k_{r}^{eI}J_{1}\left(k_{r}^{eI}a\right) & k_{r}^{eII}J_{1}\left(k_{r}^{eII}a\right) & k_{r}^{eII}N_{1}\left(k_{r}^{eII}a\right) & 0\\ 0 & J_{o}(k_{r}^{eII}b) & N_{o}\left(k_{r}^{eII}b\right) & -K_{o}\left(k_{r}^{u}b\right)\\ 0 & -k_{r}^{eII}J_{1}\left(k_{r}^{eII}b\right) & -k_{r}^{eII}N_{1}\left(k_{r}^{eII}b\right) & k_{r}^{u}K_{1}(k_{r}^{u}a) \end{array}\right]\left[\begin{array}{c} C1\\ C2\\ C3\\ C4 \end{array}\right]=0$$

Similar to Equation (A3), when the determinant of the matrix vanishes, Equation (A6) yields a nontrivial solution. Since the matrix of the particle amplitude constants (*C*1, *C*2, *C*3, and *C*4) cannot be a zero trivial solution, the matrix on the left must equal zero. Again, by using these parameters, the particle amplitude constants (*C*1, *C*2, *C*3, and *C*4) can be found and utilized to calculate the radial dependence of differential mass sensitivity (Equation (2)).
